# Sexual Dimorphism in Glucose and Lipid Metabolism during Fasting, Hypoglycemia, and Exercise

**DOI:** 10.3389/fendo.2015.00061

**Published:** 2015-04-27

**Authors:** Maka S. Hedrington, Stephen N. Davis

**Affiliations:** ^1^Department of Medicine, University of Maryland, Baltimore, MD, USA

**Keywords:** exercise, fasting, glucose, hypoglycemia, lipids, metabolism

## Abstract

Sexually dimorphic physiologic responses occur during fasting, hypoglycemia, and exercise. The areas covered in this mini review include studies that have used isotopic tracer methods and/or euglycemic clamp studies to investigate substrate metabolism during the above common physiologic stress. Women have greater reliance on lipid metabolism during fasting, hypoglycemia, and exercise while men exhibit preference of carbohydrate utilization. Plasma glucose concentrations were shown to be lower, while free fatty acids (FFA) and lipolysis higher in women compared to men after fasting. Hypoglycemia resulted in significantly reduced epinephrine, norepinephrine, glucagon, growth hormone, pancreatic polypeptide, and hepatic glucose production responses in females as compared to males. Sexual dimorphism during exercise was demonstrated by higher glycerol and FFA responses in women compared to men and higher carbohydrate oxidation rate in men. Mechanisms that can increase lipolytic rates in women include higher total fat mass, enhanced lipolytic sensitivity to epinephrine, and increased activation of β adrenergic receptors.

## Introduction

Physiologic responses to fasting, hypoglycemia, or exercise include activation of critical homeostatic (counterregulatory) mechanisms to maintain or restore euglycemia. These mechanisms include a series of autonomic nervous system (ANS), neuroendocrine and metabolic responses that increase glucose availability by stimulating initially hepatic (later renal) glycogenolysis and gluconeogenesis and enhanced lipolysis. Increased peripheral glycogenolysis provides three carbon precursors such as lactate and pyruvate for gluconeogenesis. Lipolysis generates glycerol as another important gluconeogenic substrate and free fatty acids (FFA) as energy that drives the process. Proteolysis also produces substrates (amino acids) for the gluconeogenic process. Although these are general responses in both sexes, there appears to be significant quantitative gender-based differences in substrate utilization. A number of studies have demonstrated preference of carbohydrate metabolism in men and lipid in women.

Understanding the sexual dimorphism present in fuel oxidation during different metabolic states could contribute to the development of improved and specific gender-based nutritional guidelines such as during exercise programs. For individuals with diabetes, preventive and therapeutic strategies modified for each gender could, potentially, assist in achieving and sustaining target glycemia.

In this mini review, we will discuss sexual dimorphism in glucose and lipid metabolism during different metabolic states while concentrating on *in vivo* mechanistic studies that have used isotopic tracer methods and/or hyperinsulinemic glucose clamp studies to investigate gender-based differences in substrate metabolism during fasting, hypoglycemia, or exercise.

## Fasting

General responses to fasting are gender-neutral, and include increases in plasma fatty acid and ketones, and a decrease in plasma glucose concentrations (1). However, the decrement in glucose and surge in lipids is significantly greater in women than in men.

The gender-related differences start appearing even during short-term calorie restriction and become prominent during prolonged fasting (2, 3). Mittendorfer et al. studied whole body lipid and glucose kinetics during basal (14 h) and short-term (22 h) fasting in healthy men and women matched for percent body fat (3). Isotope labeled tracers of glucose ([^2^H_2_]glucose) and glycerol ([^2^H_5_]glycerol) were used to measure substrate kinetics. The study demonstrated that women had a greater basal glycerol rate of appearance (Ra), which is an index of whole body lipolytic rate (women: 2.1 μmol⋅kg body wt^−1^⋅min^−1^ vs. men: 1.5; *p* < 0.05). Glucose kinetics (Ra and Rd) appeared to be similar between the groups. Thus, metabolic changes during basal and short term fasting involved a sexual dimorphism in lipid but not glucose metabolism. Two major regulators of lipolysis: insulin (inhibition) and epinephrine (stimulation) were suggested to be related to the reported metabolic changes. Insulin was numerically lower in women compared to men (women: 6 μU/ml vs. men: 9), while epinephrine concentration during 14–22 h of fasting was significantly higher in men (women: 57 pg/ml vs. men: 23; *p* < 0.05). Since there was no difference in glucose production between the genders, the lower insulin concentration could suggest increased hepatic insulin action in women compared to men under short-term fasting conditions.

Greater gender-based metabolic differences were demonstrated during more prolonged fasting (38–72 h) (4–7). Plasma glucose concentrations were lower after 38 h of fasting (4) (women: 3.9 mmol/l vs. men: 4.4; *p* = 0.02), while FFA (women: 1.3 mmol/l vs. men: 1; *p* < 0.02) and lipolysis (women: 259 μmol/kcal vs. men: 194; *p* = 0.004) were higher in women compared to men. About 72 h of fasting resulted in significantly higher plasma glycerol levels in women (0.2 mmol/l, *p* < 0.05) compared with men (0.1 mmol/l) (6). Merimee et al. (7) also investigated metabolic changes during 72 h fast and reported that the plasma glucose level fell to 66 mg/dl in men and 48 mg/dl in women (*p* < 0.01), and the mean peak FFA concentration was 2.3 mmol/l in women and only 1.4 mmol/l in men (*p* < 0.01). Gender-based differences in FFA kinetics were demonstrated in 106 healthy subjects (8). The study showed that total FFA Ra and FFA Ra with respect to fat-free mass were greater in women than in men. This dimorphism was explained by the differences in body composition between genders (i.e., more body fat in women).

Possible explanations for the observed gender-based differences in lipid mobilization could include sexually dimorphic changes in anatomic fat distribution (9). Men have a higher distribution of visceral fat and women have greater lower body and subcutaneous fat. Lower body fat has been demonstrated to exhibit greater lipolytic rates resulting in higher plasma FFA concentration compared to men (4, 10–12). Interestingly, visceral adipose tissue is responsible for greater hepatic FFA delivery in women than in men and the effect increases with increasing visceral fat (13). Subcutaneous fat has a greater leptin expression rate compared to visceral adipose tissue (14). Leptin is a “satiety hormone” that regulates the amount of fat stored in the body and women have been demonstrated to have two to three times higher levels compared to men (14, 15). This has led to the speculation that adipose tissue in women is somewhat resistant to leptin’s lipogenic actions. However, two other studies, both by Jensen et al. (16), demonstrated that the association between plasma leptin concentration and percent body fat was similar in both genders. Thus, the role of leptin sensitivity in men and women remains unclear.

Several studies have reported improved insulin sensitivity in women and suggested insulin’s role in sexually dimorphic metabolic changes (17–22). However, in these studies, the influence of the menstrual cycle was either not considered or women were studied during follicular phase when insulin sensitivity is increased (23, 24). Many other studies showed no difference in insulin sensitivity between genders (4, 25–31). Therefore, the effects of insulin action on gender-specific metabolic changes remain controversial.

## Hypoglycemia

The hypoglycemic clamp technique produces a standardized decrease in plasma glucose and stable glycemic plateau (32). Controlling the rate of fall of plasma glucose or the period of hyperinsulinemic euglycemia immediately before inducing hypoglycemia has been reported to affect the magnitude of neuroendocrine responses during hypoglycemia. Additionally, the magnitude of counterregulatory responses doubles for each 10 mg/dl decrement in plasma glucose below 70 mg/dl.

**Figure 1:**
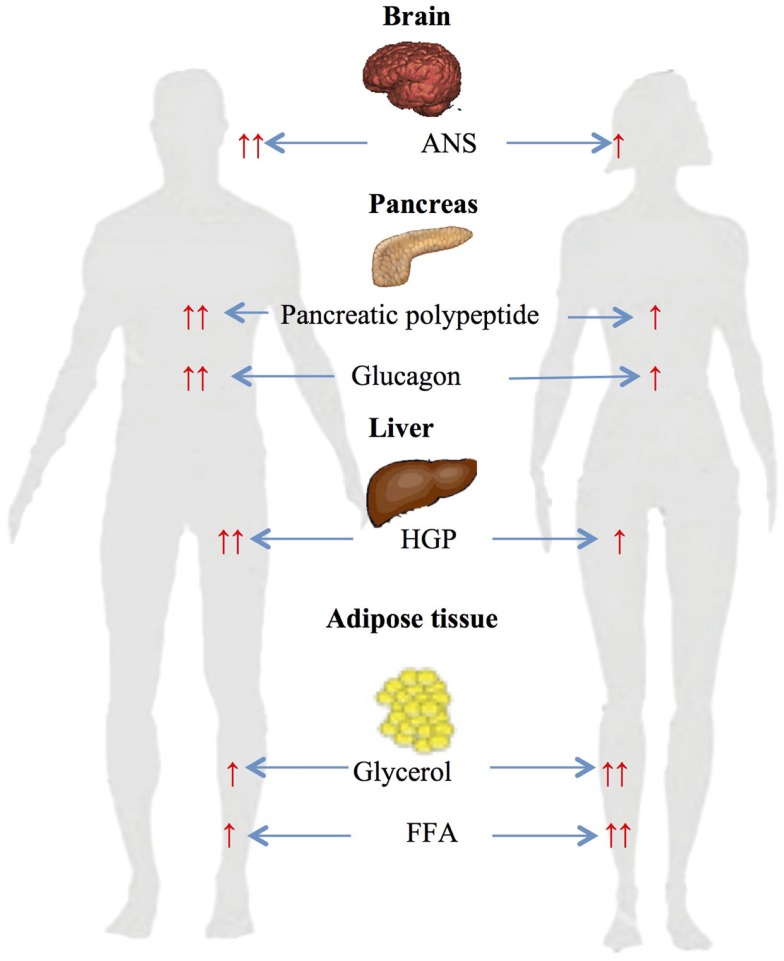
**Gender-based differences in glucose and lipid metabolism during hypoglycemia ↑ – increased, ↑ ↑ – significantly increased, ANS – autonomic nervous system, HGP – hepatic glucose production, FFA – free fatty acids**.

We have previously investigated gender-related differences in ANS, neuroendocrine and metabolic counterregulatory responses during hypoglycemia, and the physiologic mechanisms that determine those differences (33–37). We have reported that large sexual dimorphisms exist in counterregulatory responses during both a single episode of hypoglycemia and following repeated hypoglycemia (Figure 1). We have also described the role of CNS drive in triggering gender differences in metabolic responses and estrogen’s role in regulating ANS, neuroendocrine and metabolic responses to hypoglycemia.

Gender-specific changes during a single-step 2-h hyperinsulinemic hypoglycemic clamp of 50 mg/dl were investigated in age and BMI matched males and females (34). Data obtained during the steady-state period at the end of clamps demonstrated significant sexual dimorphism in neuroendocrine and metabolic responses to hypoglycemia. Epinephrine (women: 5.7 nM vs. men: 8.8; *p* < 0.05), norepinephrine (women: 2.6 nM vs. men: 3.3; *p* < 0.05), glucagon (women: 127 ng/l vs. men: 215; *p* < 0.05), growth hormone (women: 36 μg/l vs. men: 53; *p* < 0.05), pancreatic polypeptide (women: 144 pM vs. men: 291; *p* < 0.05), and hepatic glucose production (women: 9.5 μmol/kg/min vs. men: 18.1; *p* < 0.05) responses were significantly reduced in females compared to males. Diamond et al. (38) and Amiel et al. (39) have also demonstrated similar sexual dimorphisms in neuroendocrine counterregulatory responses during hypoglycemia in males and females.

It was investigated whether gender-based differences in metabolic changes observed in healthy subjects (described above) also occur in individuals with type 1 diabetes (37). The study demonstrated that women with type 1 diabetes (similar to healthy women) had significantly reduced neuroendocrine (growth hormone – women: 15 μg/l vs. men: 25; *p* < 0.05), ANS (epinephrine – women: 2.6 nmol/l vs. men: 5; norepinephrine – women: 1.7 nmol/l vs. men: 2.3; *p* < 0.05), endogenous glucose production (women: 3.9 μmol⋅kg^−1^⋅min^−1^ vs. men: 9.4, *p* < 0.01), and cardiovascular (mean arterial pressure – women: 75 mmHg vs. men: 83; *p* < 0.01) responses to moderate hypoglycemia compared to men. The above study demonstrated that significant neuroendocrine and metabolic sexual dimorphisms exist in responses to hypoglycemia in individuals with type 1 diabetes.

In searching for the cause of sexually dimorphic counterregulatory responses during hypoglycemia, several mechanisms have been proposed. One plausible explanation was that differences in the neuroendocrine responses may have been due to differential glycemic thresholds. A glycemic threshold is defined as the plasma glucose level at which counterregulatory hormones are released. Thus, 15 lean healthy men and women were studied during separate randomized single step hyperinsulinemic glucose clamp studies at euglycemia (90 mg/dl) or hypoglycemia of 70, 60, or 50 mg/dl (33). The study demonstrated that the magnitude of physiological counterregulatory responses that affect glucose and lipid metabolism were reduced in healthy women compared to men during each discreet level of hypoglycemia. However, the cause of this dimorphism did not appear to be differential glycemic thresholds as both sexes demonstrated similar plasma glucose levels at which counterregulatory mechanisms were activated (epinephrine, glucagon, growth hormone, cortisol, and pancreatic polypeptide – 70–79 mg/dl; muscle sympathetic nerve activity – 60–69 mg/dl; and norepinephrine – 50–59 mg/dl). The finding that direct sympathetic nerve activity as well as hypothalamo-pituitary responses were reduced during hypoglycemia point to a reduced central nervous system efferent response as a more likely mechanism for the sexually dimorphic counterregulatory responses occurring during hypoglycemia.

Another plausible mechanism for gender-based differences in substrate metabolism involves female reproductive hormones. Estrogen has been demonstrated to reduce lipolysis and plasma catecholamine concentration by either inhibiting secretion or accelerating degradation (40, 41). To investigate estrogen’s role in counterregulatory differences between genders during hypoglycemia, postmenopausal women on estrogen replacement were compared with postmenopausal women who did not receive replacement therapy and with age and BMI matched men during a single episode of hypoglycemia (35). Postmenopausal women on estrogen replacement were found to have reduced (epinephrine, muscle sympathetic nerve activity, pancreatic polypeptide, glucagon, endogenous glucose production, lactate, and glycerol) counterregulatory responses to hypoglycemia as compared to the similarly increased responses in women who were not on the replacement therapy and men. Thus, estrogen appears to play a significant role in the gender-based premenopausal differences in hypoglycemia-induced counterregulatory responses.

Interestingly, despite the above neuroendocrine and ANS responses, glycerol was reported to be higher in females compared to males during hypoglycemia (33, 34, 37). The differences could be attributed to a greater glycerol turnover rate due to increased peripheral fat accumulation and higher percent fat mass in females. Another reasonable explanation is higher sensitivity of the female adipose tissue to lipolytic hormones (epinephrine, norepinephrine, growth hormone). In fact, women have been shown to be more sensitive to epinephrine-induced lipolysis compared to men (42–44). Schmidt et al. investigated contribution of specific adrenergic receptor(s) in mediating gender-related differences in metabolic responses (44). The study demonstrated that infusion of epinephrine resulted in greater increases in glycerol, FFA, and palmitate Ra and Rd per kilogram body weight in women compared to men. Thus, in states of high epinephrine concentrations (e.g., hypoglycemia), women would be predicted to exhibit higher lipolysis and fat oxidation.

In addition to the gender-based differences during a single bout of hypoglycemia, we have also investigated the impact of antecedent hypoglycemia on subsequent counterregulatory responses (36). Surprisingly, women appeared to be more resistant to the blunting effects of repeated hypoglycemia on ANS, neuroendocrine and metabolic counterregulatory responses. In fact, repeated hypoglycemia produced nearly twofold greater suppressive effects on counterregulatory responses in men as compared to women. Thus, after two prior episodes of moderate hypoglycemia (50 mg/dl), the usual large sexual dimorphism in counterregulatory responses was lost during a next day episode of hypoglycemia. In other words, following day 1 hypoglycemia, counterregulatory responses were equivalent during next day subsequent hypoglycemia. This finding may help explain the finding why intensive glycemic control in younger women with type 1 diabetes results in fewer episodes of severe hypoglycemia compared to men.

## Exercise

**Figure 2:**
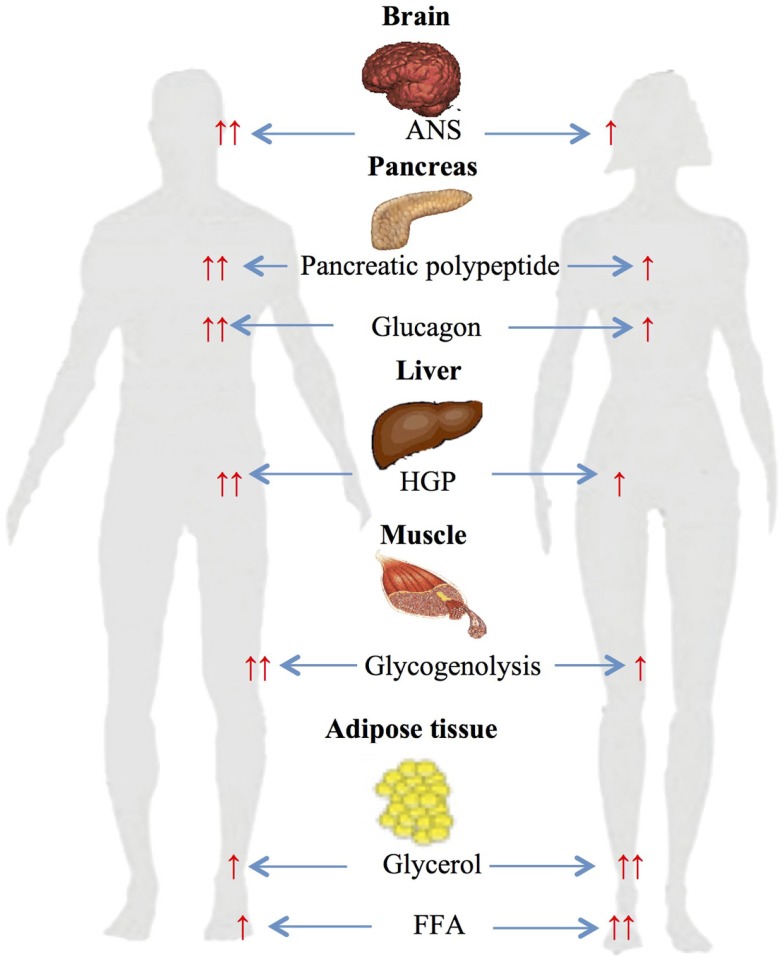
**Gender-based differences in glucose and lipid metabolism during exercise**. ↑, increased; ↑ ↑, significantly increased ANS autonomic nervous system; HGP, hepatic glucose production; FFA, free fatty acids.

A number of studies have investigated gender-based differences in metabolic changes during differing exercise work intensities (Figure 2).

Several studies from our own and other laboratories have assessed gender differences during 90 min of moderate-intensity (≈50% VO2 max) cycle ergometer exercise (45, 46). The studies demonstrated significantly higher glycerol and FFA responses in women compared to men and higher carbohydrate oxidation rate in men (45). Plasma FFA during exercise has been reported to be produced from differing anatomical sites in women and men (47–49). Roepstorff et al. demonstrated high myocellular triacylglycerol degradation in women compared to negligible in men during submaximal exercise. This was explained by higher basal intramyocellular triacylglycerol in women compared with men (48, 49). Steffensen et al. also reported similar results when measuring myocellular triacylglycerol in 21 females and 21 males during rest and exercise, and demonstrated ≈25% decrease in myocellular triacylglycerol content in females during exercise and no change in males (47).

Effects of long duration (2 h) exercise at 40% VO2max and post exercise substrate metabolism were studied in 27 healthy individuals (50). During exercise, women demonstrated higher fat oxidation (51%, *p* < 0.02) compared with men (44%). Men, on the other hand, had significantly higher circulating catecholamines and greater carbohydrate oxidation (53%, *p* < 0.01) than women (46%). There were no differences between the genders in fuel metabolism before or after (2 h recovery period) exercise. Similar metabolic differences between genders were reported in a study that investigated 7 weeks of endurance training (51). General adaptive changes to endurance training – decrease in muscle carbohydrate utilization and increase in lipid oxidation were present in both sexes; however, consistent with other exercise studies, women utilized more lipid and men increased carbohydrates during exercise. Thus duration and intensity of the exercise did not change gender-based differences in substrate metabolism during exercise.

To investigate the mechanisms for gender-specific responses during exercise, healthy women during the follicular and luteal phases of the menstrual cycle and healthy men were studied during 90-min cycling exercise at 65% VO2max (52). [6,6-2H]glucose was used to calculate glucose kinetics, which were significantly different between women in each phase of the menstrual cycle and between men and women. Women in the luteal phase had lower glucose Ra (49 μmol⋅kg^−1^⋅min^−1^; *p* = 0.03), Rd (49 μmol⋅kg^−1^⋅min^−1^; *p* = 0.03), and metabolic clearance rate (10 ml⋅kg^−1^⋅min^−1^; *p* = 0.04) at 90 min of exercise compared with women in follicular phase (52 μmol⋅kg^−1^⋅min^−1^, 52 μmol⋅kg^−1^⋅min^−1^, 9 ml⋅kg^−1^⋅min^−1^, respectively). Women, regardless of the menstrual cycle, had a lower respiratory exchange ratio, glucose Ra and Rd, and metabolic clearance rate during exercise compared with men (*p* < 0.05). During the study, muscle biopsies were taken to investigate muscle glycogen utilization. Women in the luteal phase had lower proglycogen (*p* = 0.04), macroglycogen (*p* = 0.04), and total glycogen (*p* = 0.02) use during exercise compared with women in follicular phase. Thus female reproductive hormones appear to play an important role in muscle sparing of glycogen utilization. Adding to this finding, biopsy samples from skeletal muscle, heart, and liver in estradiol treated rats demonstrated reduced glycogen metabolism compared to non-treated male rats (53, 54).

The above studies clearly demonstrate that women have reduced carbohydrate and increased lipid utilization compared to men. The sexually dimorphic changes in ANS responses appear to be a major contributor to these metabolic findings. Women during exercise had significantly lower circulating epinephrine and norepinephrine that are known to stimulate muscle glycogenolysis and carbohydrate oxidation (45, 55, 56). Second, the finding of increased lipid metabolism can be explained by sexually dimorphic stimulation of adrenergic receptors: while both adrenoreceptors (α and β) are activated in men during exercise, women have been shown to depend solely on β receptors (57, 58). Since α adrenoreceptors inhibit while β stimulate lipolysis, this would result in net greater lipolysis in women (45).

## Conclusion

Short-term fasting (14–22 h) resulted in sexually dimorphic metabolic changes, which were more pronounced during prolonged fasting (38–72 h): lipid metabolism was reported to be higher and carbohydrate utilization lower in women compared to men.

Neuroendocrine, ANS, and metabolic responses during hypoglycemia were nearly twofold greater in men compared to age matched premenopausal women. Metabolic changes during hypoglycemia were similar to fasting: women’s reliance on lipolysis was higher and carbohydrates lower. Surprisingly, women appeared to be resistant to the blunting effect of repeated hypoglycemia, which abolished the greater neuroendocrine and ANS responses usually observed in men during hypoglycemia.

Autonomic nervous system responses were increased in men relative to women across a broad spectrum of submaximal exercise intensities. Exercise intensity and duration as well as endurance training also resulted in increased carbohydrate oxidation in men but greater lipid utilization in women.

The mechanisms that trigger these differences could include: (1) sexually dimorphic fat distribution, (2) estrogen’s effect on circulating catecholamines, (3) gender-based differences in epinephrine sensitivity, and (4) differences in CNS drive in triggering gender-specific neuroendocrine, ANS, and metabolic responses.

## Conflict of Interest Statement

The authors declare that the research was conducted in the absence of any commercial or financial relationships that could be construed as a potential conflict of interest.
